# Case Report: A severe case of immunosuppressant-refractory immune checkpoint inhibitor-mediated colitis rescued by tofacitinib

**DOI:** 10.3389/fimmu.2023.1212432

**Published:** 2023-06-26

**Authors:** Mark W. D. Sweep, Martijn J. H. Tjan, Mark A. J. Gorris, Kalijn F. Bol, Harm Westdorp

**Affiliations:** ^1^ Department of Medical Oncology, Radboud University Medical Center, Nijmegen, Netherlands; ^2^ Department of Medical BioSciences, Radboud University Medical Center, Nijmegen, Netherlands; ^3^ Division of Immunotherapy, Oncode Institute, Nijmegen, Netherlands

**Keywords:** colitis, immune checkpoint inhibitor, tofacitinib, adverse event, cancer, autoimmunity, case report

## Abstract

Immune checkpoint inhibitor therapy for cancer treatment can give rise to a variety of adverse events. Here we report a male patient with metastatic melanoma who experienced life-threatening colitis and duodenitis following treatment with ipilimumab and nivolumab. The patient did not respond to the first three lines of immunosuppressive therapy (corticosteroids, infliximab, and vedolizumab), but recovered well after administration of tofacitinib, a JAK inhibitor. Cellular and transcriptional data on colon and duodenum biopsies shows significant inflammation in the tissue, characterized by a large number of CD8 T cells and high expression of PD-L1. While cellular numbers do decrease during three lines of immunosuppressive therapy, CD8 T cells remain relatively high in the epithelium, along with PD-L1 expression in the involved tissue and expression of colitis-associated genes, indicating an ongoing colitis at that moment. Despite all immunosuppressive treatments, the patient has an ongoing tumor response with no evidence of disease. Tofacitinib might be a good candidate to consider more often for ipilimumab/nivolumab-induced colitis.

## 1 Introduction

Immune checkpoint inhibitors (ICIs), such as monoclonal antibodies (mAbs) targeting programmed cell death ligand 1 (PD-L1), programmed cell death-1 (PD-1) and cytotoxic T-lymphocyte-associated antigen-4 (CTLA-4), have become a mainstay in the treatment of several cancers, and achieve their effect by restoring the immunological response against tumor cells (1). Besides the intended induction of an antitumor response, they can also elicit an immune response against healthy tissues, leading to a wide variety of immune-related adverse events (irAEs). In patients receiving CTLA-4 inhibition, the incidence of grade 3-4 toxicity is approximately 24% while with anti PD-(L)1 therapy this is around 14% (2, 3). In combination therapy the incidence of grade 3-4 irAEs even increases to 55%. Colitis is the most frequently observed grade 3-4 irAE in dual-agent checkpoint inhibition (7.7%) (4). Guidelines on immunotoxicity advise intravenous prednisone 1-2 mg/kg for grade 3-4 ICI-mediated colitis. If symptoms persevere or worsen, second line treatment with infliximab or vedolizumab should be considered (5, 6). Here we present a case of ICI-mediated colitis, refractory to prednisone, infliximab and vedolizumab, but responsive to tofacitinib, a Janus kinase (JAK) inhibitor, which was recently found to be an effective therapy for ulcerative colitis (7). Tofacitinib inhibits several subsets of JAK, by which it reduces activation of signal transducer and activator of transcription proteins, which in turn leads to reduced cytokine production, mainly within the interleukin family (8). In this way it is capable of modulating immune responses.

## 2 Case presentation

**Figure 1:**
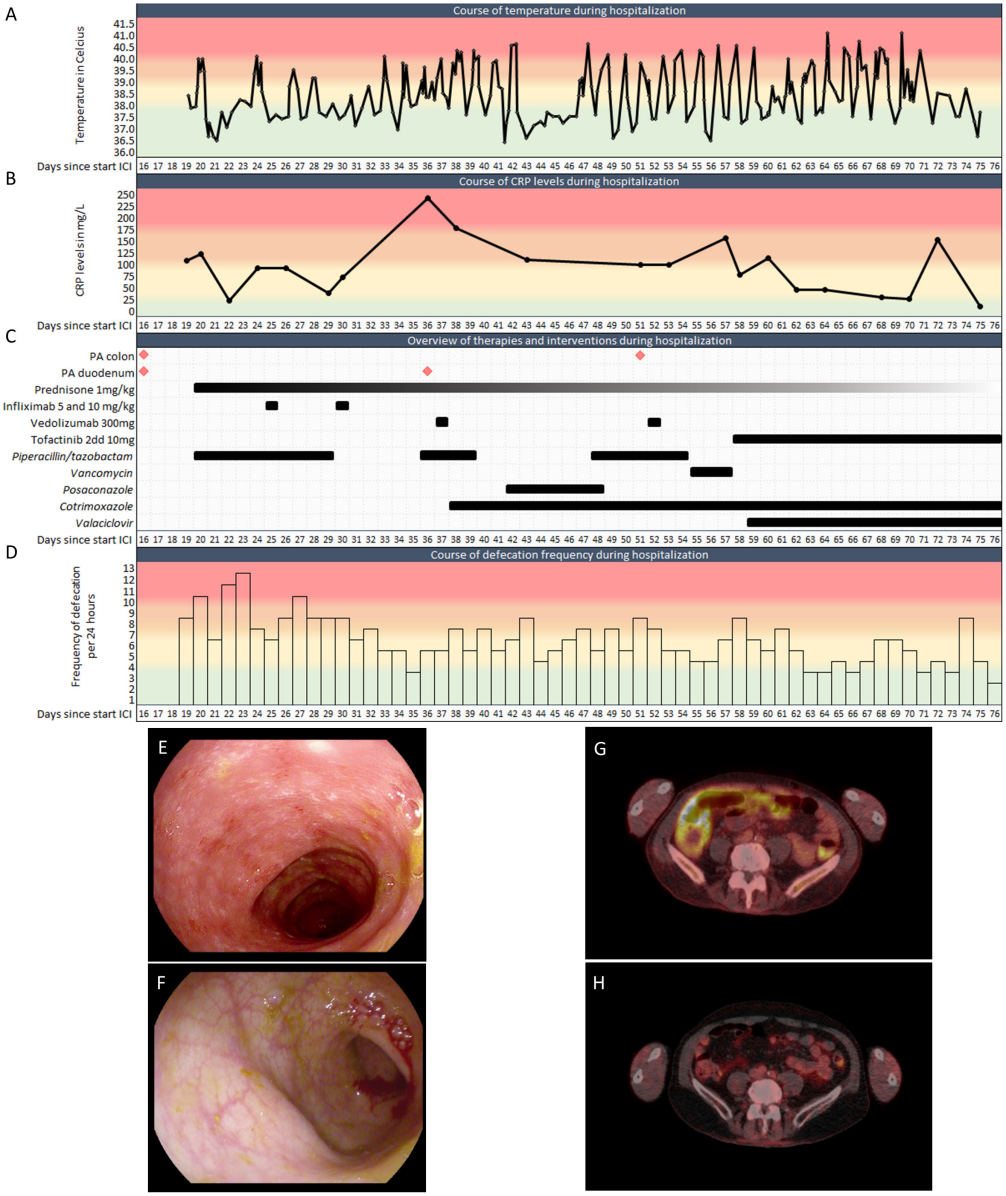
Clinical features of the patient during hospitalization. **(A)**. Temperature during hospitalization (Celsius). **(B)**. CRP levels during hospitalization. **(C)**. Biopsies, anti-inflammatory drugs and antimicrobials during hospitalization. **(D)**. Defecation frequency during hospitalization. **(E, F)**. Representative images of sigmoidoscopies at **(E)** day 16 and **(F)** day 51. **(G)**. PET-CT at day 48 with FDG-uptake in the colon. **(H)**. PET-CT 3 months after discharge showing physiological FDG-uptake, demonstrating the absence of colitis after tofacitinib. PA: Pathological assessment; CRP: C-reactive protein; ICI: Immune checkpoint inhibitor. Colors indicate severity from green to red.

A 67-year-old patient was referred to our academic hospital with BRAF-wildtype metastatic melanoma (lymph nodes, lung, liver, bone, spleen, and peritoneum). Combination immunotherapy with ipilimumab (3mg/kg Q3W) and nivolumab (1mg/kg Q3W) was initiated. At day 6 and 13 of the first cycle, the patient also received palliative radiotherapy to L2-L5 (2x 8 Gy). Shortly after, he developed watery stools, nausea, vomiting, abdominal pain, and fever. At day 16 a gastroduodenoscopy and sigmoidoscopy were performed. Macroscopically, only a mild unspecific colitis with some hemorrhoids was observed (**Figure 1E**). At day 19, microscopical results from biopsies showed strong active inflammation, along with reactive epithelium. The differential diagnosis concerned radiation-induced duodenitis, but the affected colon was not in the radiation field, and cytomegalovirus colitis was ruled out. The clinical symptoms were therefore considered as ICI-mediated colitis and duodenitis. The patient was admitted to the hospital and immunosuppressant therapy with intravenous prednisolone (1mg/kg/day) was initiated. The nausea and vomiting then disappeared. However, the fever and diarrhea persisted, including high levels of C-reactive protein (CRP), an inflammatory marker (**Figures 1A-D**). Therefore, on day 25 infliximab (5mg/kg) was added, and on day 30 another dose of infliximab (10mg/kg) was given due to the severity of symptoms. As symptoms continued not to improve, another gastroduodenoscopy was performed, still showing reactive epithelium, focal apoptosis and active inflammation in the biopsies. Therefore, vedolizumab (300mg) was given on day 37 and 52. Prior to the latter administration of vedolizumab, another sigmoidoscopy macroscopically showed a very mild colitis with some ulcers (**Figure 1F**), while microscopically a moderately active colitis with focal apoptosis was described. Unfortunately, despite 3 lines of immunosuppressive therapy, diarrhea and fever persevered and showed no sign of improvement. Therefore, after extensive deliberation in our multidisciplinary immunotoxicity meeting, and with the consulting gastroenterologist, tofacitinib (10mg orally twice daily) was started on day 58. Thereafter, defecation frequency slowly reduced, and consistency improved. The fever spikes persisted up to 10 days after the first dose of tofacitinib but diminished after that. The patient could be discharged after almost 2 months of hospitalization.

During his admission the diagnosis ICI-mediated colitis was frequently challenged due to the unresponsiveness of immunosuppressive therapies. However, aside from an intercurrent staphylococcus hominis PICC-line infection, which was adequately treated with vancomycin, no pathogens were found in multiple blood and urine cultures or stool samples. Cotrimoxazole and valaciclovir were given as Pneumocystis jirovecii pneumonia and antiviral prophylaxis, respectively. The prescribed pragmatic broad-spectrum antibiotics did not impact patient’s fever or defecation frequency and consistency (**Figures 1C, D**), further supporting the diagnosis of ICI-mediated colitis. Additionally, no alternative cause could be found on abdominal CT. A PET-CT scan at day 48 indeed showed the radiological impression of enterocolitis (**Figure 1G**). Besides, earlier FDG-avid melanoma localizations were no longer avid, suggesting a complete response to immunotherapy.

After discharge from our hospital, CRP levels quickly returned back to normal, the patient no longer had fever and his defecation pattern slowly normalized. Prednisone and tofacitinib were successfully tapered over a period of 5 months. PET-CT imaging three months after discharge showed an ongoing radiological complete remission of the colitis (**Figure 1H**) and a complete response of all his melanoma metastases. Since his single cycle of ipilimumab and nivolumab, he received radiotherapy for a solitary PET positive bone metastasis. No further disease activity was detected up to date with over two years of follow-up.

## 3 Methods

### 3.1 Patient material

Formalin-fixed, paraffin-embedded (FFPE) tissue biopsies were collected from the case patient, and from three control patients with diarrhea, without any signs of colitis in their colon biopsy.

### 3.2 RNA sequencing

RNA was isolated from FFPE tissues using the RNeasy FFPE Kit (Qiagen; 73504), according to the manufacturer’s protocol.

A total of 120 ng RNA per sample was sequenced as previously described, except with 38-bp paired-end reads rather than 50-bp (9). Alignment and counting of reads was performed using the seq2science pipeline (10).

Differential gene expression was identified using DESeq2, using the ashr method for log2 fold change shrinkage (11, 12). Results were further analyzed with clusterprofiler and enrichplot, using annotated genes from Gene Ontology (13–15). Individual gene sets of interest were retrieved from the Protein-Interaction Database and GeneRIF Biological Term Annotations, and visualized using pheatmap (16–18).

### 3.3 Multiplex immunohistochemistry

Slides were subjected to multiplex immunohistochemistry (mIHC) as previously described (19). Applied antibody panels and reagents are listed in **Supplementary Tables 1** and **2**. Slides were imaged on the Vectra 3.0.4 (PerkinElmer). Tissue images were segmented into epithelium and stroma using inForm 2.4.8 software (Akoya Biosciences). Cellular phenotyping was performed using ImmuNet (20).

## 4 Multiplex IHC and RNAseq analysis of the different biopsies

**Figure 2:**
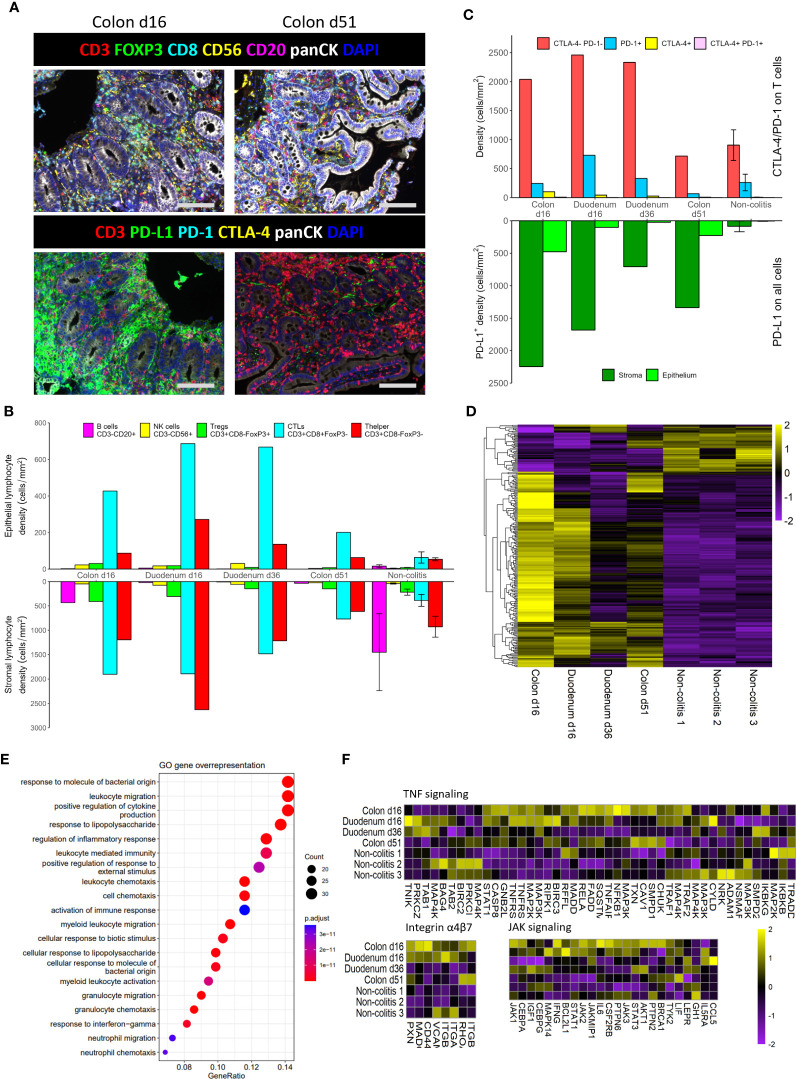
Multiplex IHC and RNAseq analysis of the different biopsies. **(A)**. Representative images of multiplex immunohistochemistry staining on colon biopsies from the patient. Scale bars represent 100 µm. **(B)**. Densities of lymphocyte subsets in the epithelium and stroma. **(C)**. Cell counts of immune checkpoint expression (CTLA-4 and PD-1) on T cells, as well as regional PD-L1 expression on all cells. **(D)**. Heatmap for Z-scores of differentially expressed genes (DEGs) of the first biopsies versus the non-colitis controls. **(E)**. Enrichment of DEGs in GO annotated pathways. **(F)**. Heatmaps for Z-scores of genes that belong to pathways that are targeted in this patient. Error bars are standard error of the mean.

We performed mIHC to study the phenotype and localization of immune cells in the patient’s colon and duodenum. Representative images showed the presence of many cytotoxic T lymphocytes (CTLs; CD3^+^CD8^+^FoxP3^-^) in the colon at day 16, compared to day 51 (**Figure 2A**). Quantification shows a dramatic increase in immune cells in the early inflamed tissue compared to non-colitis controls (**Figure 2B**). Specifically in the epithelium, the T cell response seems to be CD8 dominated. Interestingly, the density of CTLs is still high in the duodenum at day 36, after administration of high-dose prednisone and infliximab, whereas helper T cells (CD3^+^CD8^-^FoxP3^-^) have been attenuated to a greater extent. At day 51, after a first dose of vedolizumab, CTL infiltration in the colon seems attenuated, but still higher than non-colitis controls.

In addition to these immune cell subsets, we looked at expression of immune checkpoint molecules PD-1, CTLA-4, and PD-L1. The numbers of PD-1^+^ and CTLA-4^+^ T cells increase as infiltration of T cells increases (**Figure 2C**), but the relative shifts do not seem to be that large. In contrary, the number of PD-L1^+^ cells are hugely increased in the inflamed tissue, especially in the stromal compartment (**Figures 2A, C**). Interestingly, in spite of a reduction of lymphocytes at day 51 (**Figure 2B**), many PD-L1^+^ cells remain in the colon (**Figure 2C**).

Besides phenotypical characterization of immune cells, cellular signaling and activation is also an important indicator of the state in a tissue. Therefore, we sequenced RNA of the aforementioned tissues. A comparison between the two day 16 biopsies and the non-colitis controls, corrected for tissue origin, resulted in a total of 247 differentially expressed genes (DEGs) (**Figure 2D**). Curated pathways of the Gene Ontology database were then used to identify pathway enrichment based on DEGs. The top 20 enriched pathways show many pathways involved in migration and activation of immune cells, as well as a response to bacteria (**Figure 2E**).

Knowing that immune cells are highly present in the tissue and also seem activated on a transcriptional level, we were interested in the relative expression of RNA associated with pathways that were eventually targeted with infliximab (mAb against TNFα), vedolizumab (mAb against integrin α4β7) and tofacitinib (pan JAK inhibitor) (**Figure 2F**). In general, many of the genes belonging to these pathways were upregulated at day 16 versus non-colitis controls, especially in the colon. Genes seem strongly downregulated for all three pathways at day 36 in the duodenum, even though only prednisolone and infliximab have been administered by then. The colon, however, seems to have a less strong downregulation of genes (**Figures 2D, F**), as there is still some moderate to high expression of certain genes at day 51, such as *TXN*, *CAV1*, *SMPD1*, *CHUK* (TNFα), *RHOA*, *ITGB1* (α4β7 integrin), *AKT1*, *STAT3* (JAK). Thus, these particular genes are still upregulated in spite of the administration of vedolizumab on day 37.

## 5 Discussion

Here we present a patient with life-threatening ICI-mediated colitis and duodenitis, who was refractory to three lines of immunosuppressive therapy. He received prednisolone at day 19, infliximab at days 25 and 30, vedolizumab at days 37 and 52, and finally tofacitinib from day 58, after which he recovered.

In agreement with pathology assessment, we see high amounts of T cell infiltration in the patient at day 16 with mIHC. These numbers were maintained until day 36, but were moderately reduced at day 51. At the same time, PD-L1 expression was maintained at much higher levels than the non-colitis controls, even at day 51. At multiple time points, pathologists observed reactive epithelium, which may be caused by the high numbers of epithelial CTLs. These can secrete type II interferons, thereby activating and/or damaging epithelial cells (21). As a response to such pro-inflammatory cues, many cell types express PD-L1 in order to dampen inflammation. However, nivolumab targets the PD-L1 receptor PD-1 and therefore may limit the function of the PD-L1/PD-1 pathway in this case. Both high epithelial PD-L1 expression and CTL infiltration have been reported to be specific to ICI-mediated colitis compared to other gastrointestinal diseases (22, 23). Both of these features were seen in this patient. This affirms the diagnosis of ICI-mediated colitis, even though it was frequently challenged over the course of hospitalization.

On mRNA level, we found upregulation of pro-inflammatory and antimicrobial immunity pathways in the patient. During hospitalization, the patient received multiple antimicrobial agents. These agents may have affected the intestinal microbiome and immune system (21). No data on microbiome composition was available for this patient. Nevertheless, genes belonging to the anti-inflammatory drug-targeted pathways seem reduced in activity over time, perhaps indicating remission of the colitis. However, some genes that were still highly expressed at day 51 (*AKT1*, *TXN*, *CAV1*, *SMPD1*) are associated with colitis activity (24–27). Thus, the colitis was still at least moderately active on a molecular level.

Together, our data indicate a strong ongoing colitis at day 36, even though the median duration until clinical response is two weeks for infliximab (28). Therefore, infliximab seemed unsuccessful in dampening inflammation. In contrast, the severity of the colitis on a molecular and cellular level does seem reduced at day 51. This was two weeks after the first administration of vedolizumab, which is expected to show a clinical response after two or three weeks (28). Thus, vedolizumab may have been sufficient to reach clinical remission with more time. Nevertheless, clinical presentation of the patient remained worrisome, such as parental feeding dependency, fluctuations in body temperature and stool frequency. In addition, CRP levels seemed to show an initial decrease after the first dose of vedolizumab, but reached a plateau at approximately 100mg/L. Although the antimicrobial treatments may also have affected these CRP levels, it is a possible indication that vedolizumab had a reducing effect on the colitis, but could not cause further disease remission. Hence, the JAK-inhibitor tofacitinib was administered. Even though our retrospective data shows that JAK signaling was not highly active in terms of RNA expression levels at this stage, the patient’s physical condition rapidly improved on tofacitinib. Since JAK inhibition affects many cytokines and cell types, it is not clear what part of the affected signaling cascades was especially important for its effect. Its mechanism of action in immune-mediated colitis should be studied in future research. Overall, tofacitinib seemed to be beneficial for this patient.

Despite all adverse events and immunomodulatory treatments, the tumor did show a complete response to ICI treatment. Within the first year a PET/CT scan showed a solitary bone metastasis which was irradiated. Currently, more than two years after start of immunotherapy, the patient shows an ongoing response without any disease activity. However, there is an unmet need for specific treatment of steroid and infliximab-refractory ICI-mediated colitis, to avoid life-threatening cases like we described here. Tofacitinib is an interesting treatment to be considered more aptly in the future, as it has shown great effect in some patients (29–31). Yet, its safety and efficacy in this setting should be investigated.

## Data availability statement

The original contributions presented in the study are included in the article/**Supplementary Materials**, further inquiries can be directed to the corresponding author.

## Ethics statement

Ethical review and approval was not required for the study on human participants in accordance with the local legislation and institutional requirements. The patients/participants provided their written informed consent to participate in this study. Written informed consent was obtained from the individual(s) for the publication of any potentially identifiable images or data included in this article.

## Author contributions

MS and MG performed experiments. MT, KB, and HW were responsible for clinical data retrieval. MS, MT, and MG performed data analysis. MG, KB, and HW supervised this work. All authors contributed to the article and approved the submitted version.
