# Supplementary material for: Case Report: A severe case of immunosuppressant-refractory immune checkpoint inhibitor-mediated colitis rescued by tofacitinib
Source: Front Immunol. 2023 Jun 26;14:1212432. doi: 10.3389/fimmu.2023.1212432 (PMC10331137; doi:10.3389/fimmu.2023.1212432)
Supplement: Supplementary file 1 [file Table_1.docx]

Supplementary Material

Case Report: A severe case of immunosuppressant-refractory immune checkpoint inhibitor-mediated colitis rescued by tofacitinib

Mark W.D. Sweep, Martijn J.H. Tjan, Mark A.J. Gorris, Kalijn F. Bol, Harm Westdorp^*^

*** Correspondence:** Harm Westdorp; [harm.westdorp@radboudumc.nl](mailto:harm.westdorp@radboudumc.nl)

# Supplementary Tables

## Supplementary Table 1: Lymphocyte panel for multiplex immunohistochemistry

| **Order** | **Antibody target** | **Clone** | **Cat#** | **Dilution** | **Manufacturer** | **Opal dye** |
| --- | --- | --- | --- | --- | --- | --- |
| 1 | CD8 | C8/144B | M7103 | 1/200 | Agilent Dako | 690 |
| 2 | CD20 | L26 | MS-340-S | 1/600 | Thermo Scientific | 570 |
| 3 | CD3 | Sp7 | RM-9107 | 1/200 | Thermo Scientific | 520 |
| 4 | Foxp3 | 236A/E7 | 14-4777 | 1/100 | Thermo Scientific | 540 |
| 5 | CD56 | MRQ-42 | 156R-94 | 1/1000 | Cell Marque | 620 |
| 6 | Pancytokeratin | AE1/AE3+5D3 | ab86734 | 1/1500 | Abcam | 650 |

## Supplementary Table 2: Immune checkpoint panel for multiplex immunohistochemistry

| **Order** | **Antibody target** | **Clone** | **Cat#** | **Dilution** | **Manufacturer** | **Opal dye** |
| --- | --- | --- | --- | --- | --- | --- |
| 1 | PD-L1 | E1L3N | 13684S | 1/1000 | Cell Signaling | 540 |
| 2 | PD-1 | D4W2J | 86163S | 1/100 | Cell Signaling | 690 |
| 3 | CTLA-4 | BSB-88 | BSB2885 | 1/50 | Bio SB | 620 |
| 4 | CD3 | Sp7 | RM-9107 | 1/200 | Thermo Scientific | 520 |
| 5 | Pancytokeratin | AE1/AE3+5D3 | Ab86734 | 1/1500 | Abcam | 650 |
